# Dutch surveys bedpan management (1990 & 2010): progress in correct use of washer disinfectors

**DOI:** 10.1186/1753-6561-5-S6-P308

**Published:** 2011-06-29

**Authors:** GG Van Knippenberg-Gordebeke

**Affiliations:** 1 Knip con, Venlo-Boekend, Netherlands

## Introduction / objectives

In the Netherlands bedpans are regarded as semi-critical items unlike Spauldings scheme (1968), where bedpans are categorized as non-critical items. Washer-disinfectors (WD) were used incorrect for a long time. A survey in 1990 showed poor final cleaning and disinfection results. The Dutch Working Party Infection Prevention made guidelines for WD (1998), and in 2006 the International Organization for Standardization (ISO) introduced the Standard 15883 for WD:Part 3 specifies requirements intended to be used for emptying, flushing, cleaning and thermal disinfection of bedpans. I repeated the survey in the Netherlands in 2010.

## Methods

A survey was sent to Consultants Infection Prevention in 120 Dutch hospitals. The questions covered the following data: kind of bedpans, methods of emptying, cleaning and disinfection, awareness and use of national or international guidelines for WD and the validation and maintenance of WD. Final questions about the role for bedpans or WD as a source for outbreaks.

## Results

The response rate in 2010 was 77/120 hospitals (64,1%). Guidelines improved practice and manual stopped. Maintenance and validation of the WD showed good improvements. 94 % responders never searched for WD or bedpans causing outbreaks or Healthcare Associated Infections(HAI) 6,5 % reported outbreaks with the following Microorganisms: *Clostridium difficile*, *Norovirus*, *Pseudomonas aeruginosa*, *Salmonella species and Acinetobacter baumannii.* Nobody published these results or wanted to share these events.

## Conclusion

Validated well maintained WD improves patientsafety, contributes to occupational health and prevent staff from unpleasant jobs. Although bedpans can contain loads of pathogens which can be easily spread and transmit, the majority never searches for (handling) bedpans and WD as a source for HAI. More study is needed for validated data about this risk.

## Disclosure of interest

G. Van Knippenberg-Gordebeke Consultant for MEIKO washing technologies.

